# Human Cytomegalovirus as a Therapeutic Target in Glioma Stem Cells

**DOI:** 10.3390/cells15070575

**Published:** 2026-03-24

**Authors:** Tarek Bou Dargham, Eugene J. Vaios, Sean Lawler, Kristen Batich

**Affiliations:** 1 Department of Neurosurgery, Duke University, Durham, NC 27710, USA; 2 The Preston Robert Tisch Brain Tumor Center, Duke University Medical Center, Durham, NC 27710, USA; 3 Department of Radiation Oncology, Duke University Medical Center, Durham, NC 27710, USA; 4 Department of Pathology and Laboratory Medicine, Legorreta Cancer Center, Brown University, Providence, RI 02903, USA

**Keywords:** glioma, glioblastoma, glioma stem cells, human cytomegalovirus, targeted therapy

## Abstract

**Highlights:**

**What are the main findings?**

- Human cytomegalovirus preferentially localizes to glioma stem cell (GSC)-enriched compartments and associates with transcriptional and signaling networks that sustain stemness and tumor persistence.
- Viral proteins and microRNAs, including pp71, IE1, and CMV-encoded IL-10, engage pathways such as STAT3 and SOX2 to promote GSC maintenance, immune evasion, and therapy resistance.

**What are the implications of the main findings?**

- These observations support a model in which HCMV functions as an oncomodulatory driver of GSC biology and glioblastoma progression.
- Targeting HCMV within the GSC niche may represent a complementary therapeutic strategy to overcome tumor persistence and improve responses to standard glioblastoma therapies.

**Abstract:**

Glioblastoma is the most aggressive tumor among gliomas, and recurrence remains inevitable despite aggressive therapies. Resistance to existing treatment modalities is attributed in part to the presence of glioma stem cells, which comprise a distinct cell subpopulation that sustains cell renewal and tumor evasion through multiple mechanisms. Therapeutic strategies using herpesviruses have been evaluated following the discovery of differential human cytomegalovirus (HCMV) expression in glioblastoma tumor cells. The absence of expression in normal brain tissue led to multiple clinical trials demonstrating the potential clinical utility of targeted HCMV via herpesvirus-based oncolytic therapeutic strategies. This review provides a comprehensive overview of existing studies evaluating the expression and biological significance of HCMV within glioma stem cells. Targeting HCMV in this cellular compartment may disrupt the continuous cellular support and resilience of glioblastoma stem cells, thereby enhancing the efficacy of current treatments.

## 1. Introduction

Glioblastoma is the most aggressive and lethal brain tumor in adults [1,2]. Despite multimodal treatments that include maximal surgical resection, followed by radiotherapy and concomitant temozolomide chemotherapy, patients with glioblastoma have a median overall survival of less than 15 months [3] with a 2-year survival rate of 27% [4]. These poor outcomes fueled ongoing research efforts to identify the causes of glioblastoma treatment resistance. Investigations over the past two decades have attributed treatment resistance to glioma stem cells (GSCs) [5,6]. GSCs share similar characteristics with somatic stem cells, contributing to a high level of tumorigenicity and unlimited resources to sustain tumor cell growth and renewal [6]. In the hopes of targeting these cells in the future, extensive research has focused on understanding key regulators of GSC biology. Human cytomegalovirus (HCMV), first identified in glioblastoma in 2002, has been shown to enhance the stemness of GSCs, providing a rationale for developing targeted therapies that exploit this mechanism [7,8].

## 2. Glioma Stem Cells

Stem cells are independent cells that conserve themselves through self-renewal. They drive embryonic development and can transform into differentiated cells [9]. Neural stem cells, a subcategory of stem cells, are multipotent and give rise to neurons, astrocytes, and oligodendrocytes [10]. They are found in the ventricular zone and the subventricular zone, two critical regions that act as the origin of cortical neurons and glial cells [11]. These cells serve as a source of renewal and replenishment to neurons and glial cells. At the outset of cell differentiation, neural stem cells give rise to neural epithelial cells, which form the neural plate, and divide symmetrically to increase cellularity [12,13]. Neural epithelial cells further differentiate into radial glial cells, which are the primary neural progenitors of the developing human brain and provide a scaffold that guides the migration of newly generated cells from the VZ [14]. Radial glial cells serve as the primary neural stem cells during embryonic development and undergo asymmetric division to generate either a radial glial cell for self-renewal or a more fate-restricted neuronal progenitor cell. As development progresses, radial glial cells give rise to multiple neural lineages, including neurons, astrocytes, and oligodendrocytes, as well as ependymal cells that form the epithelial lining of the ventricular system and the spinal canal [15]. Throughout brain development, radial glial cells maintain a neural stem cell pool within the ventricular zone and subventricular zone. Throughout this period, the ventricular zone acts as the primary reservoir of neural stem cells, whereas the subventricular zone serves as a niche for transit-amplifying cells, which exhibit high proliferative capacity but limited self-renewal and are more fate-restricted [12,15].

### 2.1. History of Cancer Stem Cells

Tumors are organized in a hierarchical manner, and cells with stem-like properties are at the apex of this hierarchy [16]. The first cancer stem cells were isolated and purified from leukemia-initiating cells in 1997 by Bonnet et al. [17]. Neural stem and progenitor cell isolation was then achieved in 2000 via the expression of CD133 on their surface [18]. Following this discovery was the isolation of GSCs from glioblastoma specimens [19,20]. GSCs share biological characteristics with somatic stem cells, including the expression of stem-associated markers such as CD133 and ex vivo multipotency. GSC multipotency is reflected in their ability to form neurons, oligodendrocytes, and astrocytes along with glioblastoma-like tumors in culture [21]. They also exhibit the expression of other cancer stem cell markers and pathways including Sox2, Notch1, Oct4, and Nestin [22,23,24]. The expression of these markers gives them their stemness features, which include (a) self-renewal and the ability to differentiate into different lineages, thus contributing to glioblastoma heterogeneity, (b) resistance to chemotherapy and radiotherapy, and (c) high recurrence rates [16]. In xenograft studies, GSCs retain the polyclonal characteristics of the original tumor, contributing to its resistance to chemotherapy. Consequently GSCs play a critical role in tumor evasion, recurrence, and progression [25,26].

### 2.2. Glioma Stem Cells in Tumor Evasion

Glioblastoma resistance and recurrence are inevitable despite the available standard treatments. GSCs are among the major effectors underlying glioblastoma’s highly malignant characteristics [5,6]. Therapeutic resistance has been attributed to their ability to alter multiple factors including DNA repair synthesis, immunosuppression, and DNA damage response.

#### 2.2.1. DNA Repair Synthesis

Temozolomide, ionizing radiotherapy, and surgery comprise the standard of care regimen for newly diagnosed glioblastoma [27]. Temozolomide, an alkylating agent, induces cytotoxicity primarily by causing O^6^-methylguanine lesions in DNA, which depletes the repair enzyme O^6^-methylguanine DNA methyltransferase (MGMT), leading to double strand DNA breaks and tumor cell apoptosis. Radiotherapy acts synergistically via direct and indirect DNA damage, resulting in single and double strand DNA breaks. Recognition that MGMT repairs temozolomide-induced DNA damage led to the understanding that MGMT promoter methylation, which reduces its enzymatic activity, is associated with improved responses to chemotherapy [28,29]. The MGMT promoter methylation phenotype is now regarded as a prognostic factor for temozolomide response and is associated with a median increase in survival of 6.7 months in patients treated with the standard of care therapy [30]. Despite this favorable prognostic factor, nearly half of glioblastomas are MGMT unmethylated, and treatment resistance and recurrence is near-universal [31]. The high level of treatment resistance may be attributable to the presence of GSCs. GSCs have a marked increase in BCRP1 expression, which detects break point clusters, in turn increasing MGMT expression [32]. They also contribute to temozolomide resistance in tumor cells that survive initial treatment. Exposure to temozolomide has been shown to increase the proportion of stem-like side-population cells enriched in MGMT expression and drug-tolerant phenotypes, suggesting selective survival and expansion of resistant GSC subpopulations that may ultimately drive recurrence [33].

#### 2.2.2. Immunosuppression

Glioblastoma is associated with an immunosuppressed tumor microenvironment that has been characterized to alter multiple immunosuppressive factors including major histocompatibility complex (MHC) type 1, indoleamine 2,3-dioxygenase 1 (IDO1), T regulatory (Treg) cells, transforming growth factor-beta, and programmed death-ligand 1 (PD-L1) [34,35,36]. Moreover, immunosuppression is exhibited through crosstalk between GSCs and the tumor microenvironment. This helps GSCs evade the immune system and maintain the tumor’s resilience [37]. Examples of this crosstalk include GSCs recruiting M2 phenotype tumor-associated macrophages, which suppress the anti-tumor immune response [38,39,40]. GSCs also release extracellular vesicles bearing PD-L1, which suppress T-cell-mediated attack [41]. These findings suggest that GSCs may contribute to immune modulation within the tumor microenvironment through defined suppressive mechanisms, warranting further investigation.

#### 2.2.3. DNA Damage Response

Temozolomide and ionizing radiation typically cause double strand DNA breaks, leading to cytotoxic effects and cell death [42]. Bao et al. demonstrated that GSCs confer radioresistance through the preferential activation of the DNA damage response, which allows them to repair the radiation-induced DNA damage [43]. Rapolo et al. also found that CD133^+^ glioma cells exhibit enhanced survival following ionizing radiation compared to their CD133^−^ counterparts. This survival was attributed to the engagement of DNA damage repair pathways [44]. By activating DNA damage response pathways via Chk1/Chk2, NFkB, and PARP, glioma stem cells increase DNA repair capacity, which in turn undermines the efficacy of chemoradiation [44,45].

## 3. Human Cytomegalovirus in Glioma Stem Cells

Human cytomegalovirus (HCMV) is a beta herpes virus that has a seroprevalence greater than 60% in the human population. Primary infection typically occurs during childhood, and infection is mostly asymptomatic [46,47]. HCMV establishes lifelong infection characterized by latency with restricted viral gene expression and episodic replication resulting in viral shedding [48,49]. It exhibits broad cellular tropism and can infect multiple cell types, including epithelial, endothelial, and monocyte/macrophage lineage cells, through entry mechanisms mediated by distinct viral glycoprotein complexes. In particular, the gH/gL/gO complex contributes to broad infectivity, whereas the gH/gL/UL128-131 pentamer expands infection of epithelial, endothelial, and monocyte/macrophage populations. Within the central nervous system, neural progenitor cells are also permissive for HCMV infection, and viral entry into neural progenitor/stem-like cells has been shown to depend on PDGFR-α engagement by viral glycoprotein B, which promotes internalization and downstream PI3K signaling [50,51,52]. This broad tropism enables HCMV to be implicated in tumorigenesis in breast cancer, colorectal cancer, and glioblastoma [53,54,55,56,57]. Moreover, subsequent studies have identified that several oncogenic genes and proteins are expressed by HCMV, including the genes *US28* and *UL76*, as well as the immediate early protein (IE) 1 and IE2 [58,59]. In 2002, Cobbs et al. were the first to describe HCMV IE1 protein and phosphoprotein 65 (pp65) expression in malignant gliomas but not in neighboring healthy brain tissue. This discovery gave rise to a new avenue of developing highly specific and targeted treatment modalities against malignant gliomas [7]. The detection of IE1 together with structural viral proteins in glioblastoma specimens suggests that HCMV in this setting is not maintained in a strictly latent state but instead demonstrates ongoing viral transcriptional activity [7]. Limited evidence for productive viral replication suggests that HCMV infection in gliomas may resemble a persistent or restricted low-level lytic state rather than classical latency, although the balance between persistent infection and productive replication remains incompletely defined. More broadly, HCMV latency studies in non-glioma systems have shown that viral genomes can persist in a nonproductive state and reactivate in response to differentiation, inflammatory stimuli, and altered immune control [60,61,62]. Given the profoundly immunosuppressive microenvironment characteristic of glioblastoma, such conditions may facilitate episodic viral reactivation within tumor cells. The viral transcriptional state is also highly relevant for therapeutic strategies targeting CMV in glioblastoma, as antiviral agents such as ganciclovir require phosphorylation by the viral UL97 kinase, whereas immune-based approaches, including CMV-specific T-cell therapies and pp65-targeted dendritic cell vaccines, depend on presentation of viral antigens such as IE1 and pp65 [63,64,65].

Extensive research efforts have aimed to elucidate the role of HCMV in glioblastoma. Investigations have showed that HCMV has an oncomodulatory effect that promotes proliferative signaling, the evasion of growth suppression, genomic instability, interference with DNA damage responses, and escape from immune detection [62]. HCMV infection is associated with tumor progression when present at low lytically replicating levels [66]. Research over the past decade demonstrates that the expression of HCMV-specific IE1 has an oncogenic effect and promotes glioblastoma proliferation [67]. In an effort to better understand HCMV’s oncomodulatory effect and cellular distribution in glioblastoma, Fiallos et al. demonstrated that HCMV infection is preferentially maintained within GSCs. This was discovered by infecting primary derived glioma stem-like cell lines (387 and 3832) as well as standard human glioma cell lines (T98G and U87) with HCMV. The cells were then maintained in culture for five weeks without re-infection. Using immunofluorescence, they assessed the expression of IE1 proteins and quantified HCMV in infected cells. At 72 h post-infection, 387 and 3832 GSCs showed a higher percentage of IE1 positivity (70–80%) than standard glioma cell lines (~40%). This was also the case 5 weeks post-infection in 387 GSCs where they had ~40% IE1 expression on immunofluorescence compared to 20% in U87. This led to the conclusion that HCMV infection is preferentially maintained in the glioma stem cell compartment within standard glioma cell lines [8]. Taqman assays were also performed for the detection of viral DNA in GSCs versus glioma cell lines. Levels of viral DNA at five weeks were higher in GSC line 387 compared to the standard human cell line T98G [8]. HCMV’s strategic localization in GSC regions contributes to promoting stemness through the upregulation of the BMX-pSTAT3 pathway. This occurs through an increased expression of the non-receptor tyrosine kinase BMX together with enhanced phosphorylation of STAT3 secondary to infection. The activation of BMX has been linked to stimulation of the IL-6–JAK1–STAT3 signaling axis, a pathway known to regulate glioblastoma proliferation and stem-like transcriptional programs. In these models, chronic viral infection was associated with sustained STAT3 activation and the increased expression of stemness-associated genes, suggesting that HCMV can reinforce stem cell signaling networks through BMX-mediated STAT3 activation [8]. This provides a potential mechanistic link between HCMV infection and the maintenance of glioma stem cell phenotypes. This allows infected GSCs to outlive uninfected GSCs, while also upregulating aggressive stem cell markers such as SOX2, OLIG2, and C-EBPβ [8]. Interestingly, HCMV-infected glioblastoma cells exhibit slower proliferation than their uninfected counterparts, while demonstrating enhanced self-renewal capacity and resistance to temozolomide, even at a low multiplicity of infection [68]. These findings by Liu et al. underscore the important crosstalk occurring between HCMV and GSCs [68].

### 3.1. HCMV Tegument Protein, pp71

**Figure 1:**
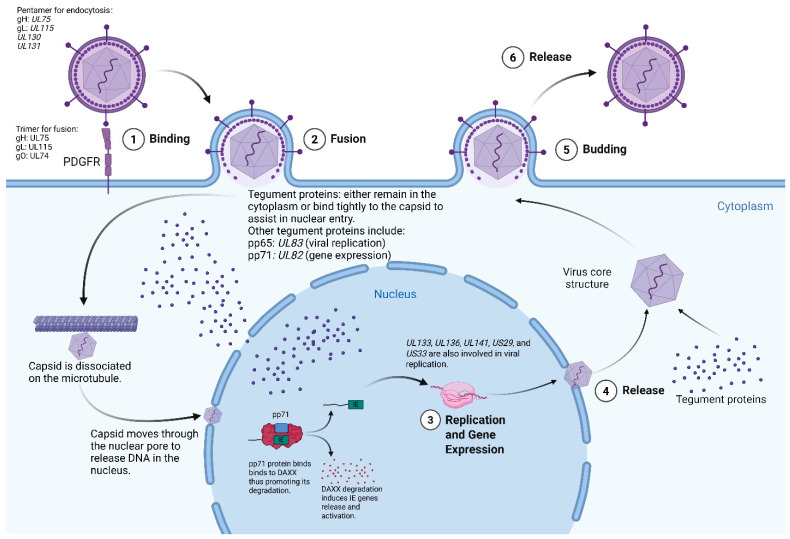
Role of different proteins and genes in human cytomegalovirus infection. gH/gL/gO trimer and the gH/gL/UL128/UL130/UL131 pentamer are crucial players in viral entry. Trimers bind to PDGFR, while pentamers bind to neuropilin-2 (Nrp-2) during entry. Abbreviations: Daxx: death domain-associated protein; PDGFR: Platelet-Derived Growth Factor Receptor. Created in https://BioRender.com.

pp71s are viral tegument proteins that are delivered into host cells upon viral entry and rapidly translocate to the nucleus, where they promote viral IE protein expression through degradation of the intrinsic antiviral restriction factor Daxx, thereby relieving transcriptional repression of viral genomes. This nuclear localization enables pp71 to initiate a viral transcriptional program [69,70]. It also degrades the hypo-phosphorylated form of retinoblastoma, a tumor suppressor protein. This promotes cell cycle progression into the S phase. Moreover, pp71 contributes to immune evasion by inhibiting the expression of MHC class I in glioblastoma [71,72,73] (Figure 1). To further investigate the role of pp71 in glioblastoma, Matlaf et al. investigated its downstream effects. Their initial findings revealed the expression of pp71 in the majority of glioblastoma cells, particularly within stem-like compartments characterized by CD133^+^ cells [74].

They also tested the influence of HCMV on the expression of the stem cell factor, a known pro-angiogenic factor in glioblastoma, whose expression is correlated with poor prognoses. Investigators found that stem cell factor expression was diminished following knockdown of pp71 in both in vitro and endogenously HCMV-infected primary glioblastoma [74,75]. This led to the conclusion that HCMV also has a pivotal role in the angiogenesis of glioblastoma. Additionally, pp71 promotes the expression of *c-myb*, which maintains and expands bone marrow and neurogenic regions of the adult brain (Table 1) [76].

### 3.2. CMV70-3P Micro Ribonucleic Acid (miRNA)

miRNAs are 20–24 non-coding RNA strands that regulate gene expression post-transcriptionally by binding to complimentary RNA sequences and forming an RNA-induced silencing complex. They play a crucial role in the maintenance of virus production and persistence by binding to mRNA [83,84,85]. Ulasov et al. [78] demonstrated that CMV70-3P microRNA regulates cancer stemness of CD133^+^ cells. Two distinct glioblastoma cell lines, namely U251 and U118, were infected with HCMV and either subjected to non-coding miRNA (NCmiRNA) as the control or CMV70-3P microRNA oligonucleotide inhibitors. The results revealed that HCMV-infected glioblastoma cell lines that were treated with CMV70-3P oligonucleotide inhibitors displayed lower neurosphere formation compared to the control group [78]. To investigate the downstream effects of CMV70-3P mRNA, SOX2 expression was measured in GSCs with and without CMV70-3P inhibitors. SOX2 is a transcription factor that is essential in maintaining stem cell pluripotency and regulating neural stem cell differentiation and homeostasis [86]. In HCMV-infected GSCs, the inhibition of CMV70-3P resulted in a 20% reduction in the SOX2 promoter as determined by qPCR. These data identify SOX2 as a functional target of CMV70-3P, implicating this viral microRNA in the modulation of GSC stemness via the transcriptional regulation of SOX2 [78].

### 3.3. Immediate Early Protein and miRNA-145

miRNA-145 is crucial in the regulation of neural stem cell fate decision and maintenance. It has been detected at low levels in GSCs, and its overexpression holds implications in halting tumor invasion and progression [87,88,89]. Soroceanu et al. [79] showed a 50–60% decrease in secondary neurosphere formation in HCMV IE1 knockdown GSC models. To further understand the downstream effects of IE1 on GSC proliferation, they measured miRNA-145 levels in three HCMV-infected GSC lines, detecting a 2.5–2.7-fold decrease in its expression. This effect was attenuated in GSC lines infected with IE1-knockdown HCMV. These findings provide evidence that IE1 promotes neurosphere formation and regulation in GSCs [79]. Moreover, these investigators showed that attenuation of HCMV IE proteins promotes apoptosis induction, the inhibition of cell cycle progression, and mesenchymal and proinflammatory phenotypic shift in HCMV-positive GSCs [79]. Fornara et al. also hypothesized that HCMV infection and the maintenance of GSCs are interconnected. Their study aimed to investigate the co-expression of IE protein and stem cell markers and their impact on patients’ overall survival. Using flow cytometry, they assessed the presence of CD133 and IE proteins in fresh surgically excised tissue from 21 patients. Their results revealed that 20 out of 21 fresh surgically excised glioblastoma tissues co-expressed CD133 and IE proteins. Nevertheless, patients who had a higher rate of HCMV-IE and CD133 co-expression had shorter overall survival [90]. While these findings support an association between HCMV infection and glioma stem-like populations, the relatively small cohort size warrants cautious interpretation. Larger, multi-center studies will be required to confirm the prevalence and biological significance of CD133–HCMV co-expression in glioblastoma.

### 3.4. STAT3

STAT3 expression and activation is a key factor for cell proliferation and the phenotypic expression of glioblastoma [91]. It is also critical for the maintenance of GSC pluripotency. Nevertheless, STAT3 and p-STAT3 were highly expressed in glioblastoma compared to normal brain tissues [92,93]. HCMV was also found to upregulate the expression of STAT3 in neural stem cells harvested from subventricular and sub granular zones infected with HCMV in murine models. The stimulation of patient-derived glioblastoma neurospheres both in vivo and in vitro was absent in the setting of knocked-down STAT3. This suggests that HCMV contributes to the proliferation of glioblastoma through the stimulation of neural stem cell-derived STAT3 [80].

### 3.5. CMV IL-10

Monocytes account for 30–50% of the glioblastoma microenvironment [94]. They regulate several functions relevant to tumor growth, including phagocytosis, angiogenesis promotion, lymphocyte recruitment, and the secretion of tumoricidal mediators [95]. Thus, they are considered important regulators of cancer progression. Monocytes can differentiate into tumor-associated macrophages and dendritic cells, allowing for a more permissive tumor microenvironment [96]. Not only can HCMV promote cancer cell proliferation through GSCs, but it also exhibits immunosuppressive effects by targeting the host immune system with a predilection for infecting monocytes. By tracking the expression of CD11b and CD45, Dziurzynski et al. investigated pp65 expression among monocyte lineages and lymphocytes using flow cytometry. Their results revealed that pp65-positive cells were predominantly detected within macrophages and microglia rather than T and B lymphocytes. Subsequent investigations of HCMV activity in GSCs showed that GSCs secrete viral IL-10 (CMV IL-10) at levels ranging from 5.62 to 111.11 pg/mL per 10^6^ per day without significantly altering endogenous human IL-10. These finding underscore the interplay between HCMV and the immune system through the secretion of an inhibitory cytokine, CMV IL-10. Notably, CMV IL-10 also promotes an immunosuppressive phenotype and induces the expression of IE1 in monocytes. These immunosuppressive effects promote the expression of monocyte inhibitory signal B7-H1 while downregulating MHC-II and CD86 stimulatory signals, in turn restricting monocytes to a naïve, immunosuppressed state and blocking differentiation into macrophages and microglia [82].

### 3.6. Other Cancer Stem Cell Markers

CD133 is a pentaspan membrane-bound glycoprotein. It was first described in mice in neuroepithelial stem cells, and later identified in tumor-initiating cells in the brain [97,98]. Notch 1 is part of the notch1-4 cognate receptors to DLL1, DLL3, DLL4, and Jagged ligands (JAG1 and JAG2). When an interaction occurs between the ligand and the notch receptors, the receptor is cleaved and downstream signaling in the nucleus takes place to induce a notch gene expression profile. These notch target genes are key regulators of cell fate, differentiation and cell cycle progression, and survival [99]. SOX2 plays a key role in the regulation of undifferentiated embryonic stem cells and is a marker associated with stemness in cancer stem cells. It is co-expressed with CD133 and regulates tumorigenicity in CD133^+^ glioblastoma [100]. Oct-4 is associated with tumor-initiating cells, but is also expressed in embryonic stem cells and germ cells. It functions as an effector of stem cell pluripotency, self-renewal, and cell differentiation [101]. Nestin is a neural stem cell marker expressed during the development of neural stem cells. It is associated with clinical outcomes in several malignancies such as colorectal cancer, prostate cancer, and glioblastoma. Its presence is associated with a cancer stem cell phenotype [102,103].

The expression of these markers was investigated to better understand HCMV’s contribution to the stemness of GSCs. Primary glioblastoma specimens were cultured under adherent and non-adherent conditions and infected with HCMV. The tissues were then assessed for neurosphere formation, a unique behavior of GSCs under non-adherent conditions. The results showed that the expression of CD133, Notch 1, Sox-2, Oct-4, and Nestin was upregulated in HCMV-infected glioblastoma cells compared to the uninfected glioblastoma cells. Nevertheless, HCMV-infected cells were able to contain neurospheres in an undifferentiated state, which can transform in the future into astrocytic and neural lineages [90].

While several studies report that HCMV infection is associated with the increased expression of stemness-associated markers and enhanced stem-like phenotypes in glioma cells, the precise mechanisms underlying these effects remain incompletely defined. In particular, it remains unclear to what extent these findings reflect the direct induction of stemness programs by viral gene products versus the preferential infection or persistence within glioma cell populations that already possess stem-like features. Nonetheless, available evidence supports the view that HCMV infection can promote or reinforce stemness-associated signaling in glioblastoma, including pathways involving STAT3 and Notch signaling [8,79,104]. It is important to note that many of the reported associations remain largely correlative rather than mechanistically defined. Several viral proteins and signaling pathways, including IE proteins, STAT3 activation, and viral cytokine homologs, have been linked to enhanced proliferation, survival signaling, immune evasion, and stemness-associated transcriptional programs in glioblastoma models. However, in many cases the precise molecular mechanisms through which HCMV directly induces or sustains glioma stem cell phenotypes remain incompletely understood. This limitation represents a significant challenge in the field and highlights the need for future studies aimed at defining the causal signaling and epigenetic pathways linking viral gene expression to glioma stem cell maintenance and plasticity.

## 4. Presence of HCMV in Glioblastoma: Conflicting Evidence

HCMV in glioblastoma remains controversial. While several studies have reported the detection of HCMV nucleic acids and proteins in glioblastoma specimens, others have failed to identify viral DNA, RNA, or protein using molecular or immunohistochemical approaches. Yamashita et al. reported no detectable HCMV DNA in glioblastoma samples using quantitative PCR [105]; similarly Holdhoff et al. found no evidence of HCMV in glioblastoma or other high-grade gliomas using a multimodal detection strategy including PCR, immunohistochemistry, and in situ hybridization [106]. Importantly, several factors may contribute to these discordant findings beyond the simple absence of virus. HCMV has frequently been reported at very low copy numbers in glioblastoma tissues, which can make detection highly dependent on assay sensitivity and experimental conditions [7,62]. In addition, studies using immunohistochemistry and in situ hybridization have suggested that HCMV infection may be restricted to specific subpopulations of tumor, including glioma stem-like cells, which could limit detection when bulk tumor tissue is analyzed [8,79]. Methodological factors may also contribute to negative results. Differences in tissue preservation and quality (i.e., predominantly necrotic tumor tissue), nucleic acid extraction efficiency, antibody specificity, and the viral gene targets analyzed can substantially affect detection sensitivity.

## 5. Targeting HCMV in Malignant Glioma

**Figure 2:**
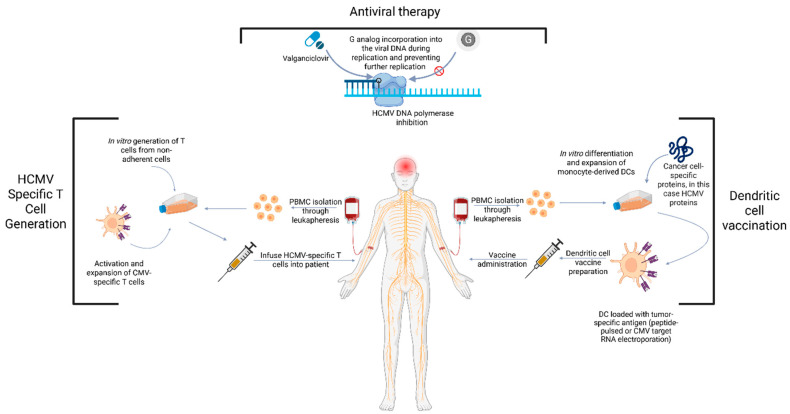
Multimodal strategies targeting HCMV in glioblastoma. Adoptive T-cell therapy, antiviral treatment, and dendritic cell vaccination. G: guanine, HCMV: human cytomegalovirus, PBMC: peripheral blood mononuclear cells. Created in https://BioRender.com.

Following the discovery of HCMV in glioblastoma in 2002 [7], accumulating evidence suggests a role for HCMV in promoting glioblastoma cell proliferation and tumorigenesis, supporting the rationale for targeting HCMV as a therapeutic strategy in glioblastoma. Accordingly, multiple treatment modalities have been explored or are currently under investigation, including antiviral therapy, dendritic cell vaccination, and adoptive CMV-specific T-cell therapy (Figure 2) [107,108,109,110].

### 5.1. Antiviral Therapy

HCMV-targeted antiviral therapy has been explored with valganciclovir [111], a prodrug of ganciclovir [112]. Both valganciclovir and ganciclovir have a safe profile, as they rarely cause any adverse effects [113]. In 2006, a randomized study evaluated the addition of valganciclovir to the standard of care for patients with glioblastoma. While there was no difference in overall survival or progression-free survival [111], post hoc analysis revealed that patients receiving antiviral therapy for more than six months had significantly improved survival (OS = 24.1 months; 95% CI, 17.4–40.3) compared to patients who received 0–6 months of antiviral therapy (OS = 13.7 months; 95% CI, 6.9–17.3, *p* = 0.0031) [111]. Following these findings, several clinical trials were conducted to assess the efficacy of ganciclovir in patients with glioblastoma. A meta-analysis integrating five randomized controlled trials demonstrated that ganciclovir-containing regimens were associated with improved overall survival compared to control arms in both newly diagnosed (OR 1.48, 95% CI 1.08–2.02, *p* = 0.014) and recurrent glioma (OR 6.51, 95% CI 2.79–15.22, *p* = 0.000015) [114,115,116]. Despite these encouraging results, understanding the mechanisms of resistance to antiviral therapy in glioblastoma remains critical for optimizing treatment outcomes. In HCMV infection more broadly, resistance to ganciclovir and valganciclovir is classically mediated by mutations in the viral UL97 kinase, which impairs phosphorylation and the activation of the drug, and less commonly by mutations in the viral UL54 DNA polymerase, which may also confer cross-resistance to other antivirals [117]. Moreover, inflammatory and immunosuppressive signals within the glioblastoma microenvironment, corticosteroid exposure, and therapy-associated immune suppression may promote heterogeneous viral reactivation and thereby create fluctuating antiviral susceptibility across tumor cell populations. Together, these considerations suggest that both standard viral resistance mutations and microenvironment-driven variability in viral transcriptional state may influence responses to valganciclovir in glioblastoma [118,119,120]. These studies provide preliminary evidence suggesting the potential therapeutic role of targeting HCMV in glioblastoma. Future work should ascertain the biological mechanisms whereby HCMV mediates gliomagenesis and therapeutic resistance to provide a rationale for drug development.

### 5.2. HCMV-Specific Dendritic Cell Vaccination

Dendritic cell vaccination has demonstrated promising results in glioblastoma outcomes. The uniquely specific expression of HCMV proteins in glioblastoma cells makes it an ideal target for immunotherapies [7,121]. ATTAC (NCT00639639) was the first CMV pp65 RNA-pulsed dendritic cell vaccine study in newly diagnosed patients with glioblastoma. This was a small randomized two-arm study whereby patients received a pp65-dendritic cell integrated with adjuvant temozolomide following standard of care resection and chemoradiation. Twelve patients were randomized to dendritic cell vaccine site pre-conditioning with either tetanus–diphtheria toxoid as a recall antigen or an autologous dose of unpulsed dendritic cells. Patients randomized to tetanus–diphtheria pre-conditioning demonstrated superior vaccine migration to draining lymph nodes, which was strongly associated with extended patient progression-free survival and overall survival [64]. A subsequent single-arm pp65-dendritic cell study (ATTAC-GM; NCT00639639) delivered pp65-dendritic cell vaccines containing the granulocyte macrophage colony stimulating factor amidst serial cycles of dose-dense temozolomide to patients with newly diagnosed glioblastoma. Although patients in this study reached superior outcomes (median overall survival of 41.1 months; median progression-free survival of 25.3 months), robust initial pp65-specific T-cell responses with serial vaccination were abrogated following the resumption of dose-dense chemotherapy [108]. These findings highlight the importance of treatment timing when combining HCMV-directed immunotherapy with temozolomide. Temozolomide induces lymphopenia and can suppress effector T-cell responses, particularly with dose-dense schedules, which may limit the durability of vaccine-induced immunity. At the same time, transient lymphodepletion may create a window for immune reconstitution and antigen-specific expansion, indicating that the relationship between temozolomide and immunotherapy is schedule-dependent. Collectively, available data suggest that CMV-directed dendritic cell vaccination may be more effective when coordinated with periods of relative immune recovery and may be adversely affected by prolonged concomitant dose-dense temozolomide exposure [108,122].

Follow-up data from these two trials reveal that nearly one-third of patients receiving CMV pp65-specific dendritic cell vaccines became long-term survivors, free of tumor recurrence for at least five years [123]. This led to a recently completed multi-institution randomized phase II study to evaluate the efficacy of CMV-specific dendritic cells compared to the control PBMC (NCT02465268). The significant findings from these early trials underscore the importance of HCMV and its potential as a therapeutic target. The optimal sequencing of CMV dendritic cell vaccine with standard of care anti-neoplastic therapy is an active area of investigation.

### 5.3. HCMV-Specific Adoptive T-Cell Therapy

HCMV specific adoptive T-cell therapy (ACT) is another immunotherapeutic approach used to target HCMV proteins in glioblastoma and simultaneously activate macrophages, cytokines, NK cells, and memory T cells. This technique has been tested in several clinical trials and demonstrates encouraging outcomes [64,109,124,125]. Crough et al. observed that CD8^+^ T-cells isolated from HCMV-seropositive glioblastoma patients exhibited impaired polyfunctional activity ex vivo following stimulation with HLA class I-restricted HCMV peptide epitopes. However, in vitro stimulation with patient-matched HCMV peptide epitopes derived from the immunodominant viral antigens pp65 and IE-1 in the presence of IL-2 restored cytokine production and cytolytic function in the CMV-specific CD8^+^ T-cells [126]. Complementing these findings, Nair et al. showed that CMV pp65-specific CD8^+^ T cells, expanded in vitro through co-culture with autologous dendritic cells pulsed with pp65 RNA, were capable of recognizing and lysing autologous primary glioblastoma tumor cells in an antigen-specific, HLA-restricted manner [65]. Khanna et al. then demonstrated the efficacy and safety of ACT in patients with recurrent glioblastoma in a phase I clinical trial, which recorded prolonged overall survival [127]. These results [125] were the rationale for a trial which evaluated ACT in patients with newly diagnosed glioblastoma. CMV-specific T cells for ACT were generated from 25 participants. The T cells were composed of a median of 79.3% CD3^+^CD8^+^ T cells and 12.8% CD3^+^CD4^+^ cells. Patients received two to six doses of ACT depending on the availability of the cells. The outcomes showed a median progression-free survival and overall survival of 10 and 21 months, respectively. Subgroup analysis showed that patients who initiated ACT before progression of the disease had a significantly better overall survival than those who took the treatment after progression (23 months and 14 months, respectively; *p* = 0.018) [125]. Similarly, in a trial of 17 patients with newly diagnosed glioblastoma, Reap et al. demonstrated that combining pp65-specific dendritic vaccination with HCMV ACT using pp65-specific T cells enhanced polyfunctional responses in vivo. Patients were randomized to ACT plus CMV pp65-specific T cells with CMV-dendritic cell vaccination or saline. Patients receiving CMV-ACT-dendritic cell vaccination had a significant increase in IFNγ+, TNFα+, and CCL3^+^ polyfunctional CMV-specific CD8^+^ T cells, and these increased polyfunctional T cell responses were associated with patients’ overall survival (R = 0.7371, *p* = 0.0369) [124]. Although these studies suggest improved outcomes in select patients, none address the underlying mechanism linking HCMV and GSCs, which are essential for glioblastoma progression and proliferation. Understanding this interaction is essential for determining whether targeting HCMV can meaningfully disrupt glioma stem cell-mediated tumor maintenance and resistance.

## 6. Conclusions and Perspectives

This review highlights the growing evidence underlying the interaction between HCMV and GSCs, a tumor cell subpopulation central to glioblastoma resistance, recurrence, and progression. Accumulating data indicate that HCMV is present within glioblastoma tissue and preferentially localizes to and persists within GSC-enriched compartments, where it modulates stemness, survival, immune evasion, and therapeutic resistance. Mechanistic studies demonstrate that HCMV gene products and regulatory elements including pp71, IE proteins, viral microRNAs, STAT3 signaling, and CMV IL-10 collectively promote self-renewal, maintain an undifferentiated state, and enhance resistance to stress in GSCs. These viral programs also reshape the tumor microenvironment through immunosuppressive mechanisms, including the modulation of myeloid cell phenotypes and the inhibition of effective anti-tumor immune responses. Importantly, several of these effects are observed even at low levels of viral infection, suggesting that HCMV exerts disproportionate biological influence within glioblastoma despite limited viral burden. These findings suggest that HCMV, unlike standard oncogenic viruses such as Epstein–Barr virus or human papillomavirus, primarily functions as an oncomodulatory virus that influences tumor progression and cellular plasticity within the glioblastoma microenvironment. Clinical investigations targeting HCMV have reinforced its potential therapeutic relevance. Antiviral therapy, dendritic cell-based vaccination, and CMV-specific T-cell approaches each demonstrate potential clinical utility, particularly in recurrent glioblastoma, underscoring the translational potential of targeting viral components expressed within tumor and stem-like cell populations. However, current therapeutic strategies largely focus on tumor-wide viral targeting rather than specifically exploiting expression within glioma stem cells. Future therapeutic efforts should move beyond viral detection alone and prioritize mechanism-driven strategies that disrupt HCMV-dependent stemness programs within GSCs. Rational approaches may include targeting viral regulators of cell cycle control, immune evasion, and stem cell signaling, as well as integrating antiviral or immune-based therapies with standard of care regimens to prevent GSC-mediated recurrence. Given the central role of GSCs in glioblastoma treatment failure, targeting HCMV within this compartment represents a highly specific and potentially transformative strategy. Continued mechanistic investigation and carefully designed clinical trials will be essential to determine whether disrupting HCMV–GSC interactions can meaningfully alter the natural history of this aggressive disease.

## Figures and Tables

**Table 1:** Characterization of HCMV molecular entities: targets and downstream outcomes in glioma stem cells. CMV IL-10: cytomegalovirus-encoded interleukin-10; Daxx: death domain-associated protein; GSC: glioma stem cell; IE1: immediate early protein 1; MHC: major histocompatibility complex; NSC: neural stem cell; PD-L1: programmed death-ligand 1; pp71: phosphoprotein 71; Rb: retinoblastoma protein; SCF: stem cell factor; SOX-2: SRY-box transcription factor 2; STAT3: signal transducer and activator of transcription 3; c-Myb: cellular myeloblastosis transcription factor.

HCMV Molecular Entity	Target	Outcome
pp71 [70,71,72,73,74,75,76,77]	Daxx	Promotes expression of IE1 protein
	Hypo-phosphorylated Rb	Promotes cell cycle progression into S phase
	MHC-1	Facilitates immune evasion
	SCF	Promotes tumor angiogenesis
	*c-myb*	Expands bone marrow and neurogenic regions in brain
CMV70-3P [78]	SOX-2	Maintains stem cell pluripotency and NSC differentiation and integrity
IE1 [79]	miRNA-145	Promotes tumor invasion and progression
IE2 [62,79]	p53, Rb	Modulates cell cycle control and promotes viral and host gene expression programs
HCMV [80]	STAT3	Promotes GSC pluripotency
US28 [62,81]	STAT3, VEGF	Promotes proliferation, angiogenesis, tumor-associated inflammation
CMV IL-10 [82]	Monocytes	Induces expression of IE1 on monocytes, thus increasing expression of B7-H1 (PD-L1), and downregulates MHC-II and CD86

## Data Availability

No new data were created or analyzed in this study.

## Abbreviations

ACT	Adoptive cell therapy
GSCs	Glioma stem cells
HCMV	Human cytomegalovirus
IDO1	Indoleamine 2,3-dioxygenase 1
IE	Immediate early
JAG1	Jagged 1
MHC	Major histocompatibility complex
MGMT	O6-methylguanine-DNA methyltransferase
miRNA	MicroRNA
ncmiRNA	Non-coding miRNA
PD-L1	Programmed death-ligand 1
pp65	Phosphoprotein 65
Treg	Regulatory T cell
